# The Reconstruction of Probation

**Published:** 1919-03-29

**Authors:** 


### THE RECONSTRUCTION OF PROBATION.

The final answer to my inquiry whether or not nursing is a profession has a direct and immediate bearing on the present unrest—an unrest which is not confined to this country, but which extends over America and the colonies. It is of supreme importance that this movement should be regarded as a whole, and especially with a view to the future. Everywhere there is a tendency, which is not unnatural, to look at the matter locally, and to apply a local and temporary remedy. If the nurses of a particular hospital become restive and discontented, or if probationers of high quality are slow in volunteering, the common method is to patch up the trouble by the agency of a few pounds. From the standpoint of the hospital this plan succeeds fairly well, but it will not prove to be more than temporarily useful, nor will it in any respect promote the interests of the nurse Students are not drawn to the professions by a small financial inducement during their period of training, and then utilising and exploiting them as toilers rather than as students. Hence the emancipation of the nurse, if it is to be effected, must be so designed that the probationer shall be regarded as a student, and the trained nurse provided with a career which will at once satisfy her ambitions and assure her of a competence during service and after.

### The Nurse's Widening Field.

The issues at stake are very great. Scientific knowledge is increasing day by day, and if the thraldom of the nurse continues it is obvious that she must be debarred from participation therein, and her sphere of influence accordingly limited. On the contrary, if her education advances automatically with the world's progress, as a doctor's education advances, her field of operations must widen and her commercial value will rise. With a wider conception of the nation's duty towards the health of the community, there must come an immensely increased demand for women workers educated and trained, not only in nursing, but in many of the sciences appertaining to health. The industrial population is woefully ignorant concerning health matters—beginning with baby's food and clothing, and including everything afterwards of which human life consists. The field is without limit, but, apparently, as it ever was, "the labourers are few."

### Looking Ahead.

What is essential for those in whose hands lies the destiny of the nurse is that they should take a detached and comprehensive view of the whole situation. Efficient organisers and administrators are useful and necessary in their sphere, but other qualities and attributes are required for this problem. In the world of commerce there are men of ability and industry who pursue the even tenour of their way, year in and year out, slowly and steadily growing. But there is also the man of enterprise who possesses the rare talent of accurately forecasting the future, and he buys in anticipation of markets that will open months from hence. If the promoters of the nurse's interests have the wit and the enterprise to act now with wisdom and without fear she will need no help from trade unions. In the most natural way she will come into her inheritance.

### Beginning with the Probationer.

The person with whom a beginning must be made is the probationer, and she must be regarded as a student, not one on whose cheap labour the hospital mainly depends for its support. In America an immense effort is being made to introduce an eight-hour day and a fifty-two-hour week for probationers, without making any claim for a corresponding reduction of the hours of "graduate" nurses, and where this has been tried a better educated and higher class of candidate have presented themselves. Miss Gladys Leigh inquires if I have counted the cost to the probationer of treating her purely as a student. I have not, and the reason therefor is that there is a tremendous wall of prejudice against admitting that a nurse probationer can begin her training under the age of two or three and twenty, when she is a grown woman. My object is to break this down if I can. As long as people say and affirm that the thing is impossible, and that all the authorities have said the last word on the subject, there is not much to be gained by discussing details, even such important details as this. Once the principle of early entrance and student probation is admitted it is extremely probable that some hospital committee will be found willing to try the experiment, and pupils will be forthcoming with suitable means at their disposal.

I deny that the nurse is an exception to all other classes of trained workers, and that it is necessary that she should be five years behind the rest when she begins her career. The whole of the argument, in my humble opinion, is built upon the principle that a probationer must be able to work for twelve hours a day for seven days a week, and without one shred of regard for the well-being of the worker now or in the future. The principle is a despicable one, the more so as it is clothed in a gaberdine of charity. Moreover, it is an ignorant principle. A report published by the Ministry of Munitions proves that the reduction of the workers' hours improved their health and increased their output. A reduction of nurses' hours, an increase of her pay, and a bright life will, without doubt, contribute to the welfare of their patients.

